# Occupational Exposure to Crystalline Silica Dust in the United States, 1988–2003

**DOI:** 10.1289/ehp.7384

**Published:** 2004-12-06

**Authors:** Abdiaziz Yassin, Francis Yebesi, Rex Tingle

**Affiliations:** ^1^Directorate of Evaluation and Analysis, Office of Evaluations and Audit Analysis, and; ^2^Directorate of Cooperative and State Programs, Office of Outreach Services and Alliances, Occupational Safety and Health Administration, U.S. Department of Labor, Washington, DC, USA

**Keywords:** crystalline silica dust, industries, occupations, OSHA IMIS, silica exposure

## Abstract

The purposes of this study were *a*) to summarize measurements of airborne (respirable) crystalline silica dust exposure levels among U.S. workers, *b*) to provide an update of the 1990 Stewart and Rice report on airborne silica exposure levels in high-risk industries and occupations with data for the time period 1988–2003, *c*) to estimate the number of workers potentially exposed to silica in industries that the Occupational Safety and Health Administration (OSHA) inspected for high exposure levels, and *d*) to conduct time trend analyses on airborne silica dust exposure levels for time-weighted average (TWA) measurements. Compliance inspection data that were taken from the OSHA Integrated Management Information System (IMIS) for 1988–2003 (*n* = 7,209) were used to measure the airborne crystalline silica dust exposure levels among U.S. workers. A second-order autoregressive model was applied to assess the change in the mean silica exposure measurements over time. The overall geometric mean of silica exposure levels for 8-hr personal TWA samples collected during programmed inspections was 0.077 mg/m^3^, well above the applicable American Conference of Governmental Industrial Hygienists threshold limit value of 0.05 mg/m^3^. Surgical appliances supplies industry [Standard Industrial Classification (SIC) 3842] had the lowest geometric mean silica exposure level of 0.017 mg/m^3^, compared with the highest level, 0.166 mg/m^3^, for the metal valves and pipe fitting industry (SIC 3494), for an 8-hr TWA measurement. Although a downward trend in the airborne silica exposure levels was observed during 1988–2003, the results showed that 3.6% of the sampled workers were exposed above the OSHA-calculated permissible exposure limit.

Silica is a mineral compound made up of one silicon atom and two oxygen atoms (SiO_2_). It has a melting point of 1,600°C and is a colorless, odorless, and noncombustible solid [American Conference of Governmental Industrial Hygienists (ACGIH) 2001]. Crystalline silica is formed when silica molecules are lined up in order and in crystal form. It is an abundant mineral in rock, sand, and soil. Quartz is a term often used to refer to crystalline silica dust.

Crystalline silica has been used in many industries such as blast furnaces, cement manufacturing, glass and concrete mixing product manufacture, ceramics, clay, glass and china pottery, electronic, foundry, sand-blasting and manufacturing abrasives, and many construction activities (Altindag et al. 2003; Flanagan et al. 2003; Irwin 2003; Rappaport et al. 2003). It is used as an abrasive agent in many industrial applications. Occupations having a high potential for exposure to crystalline silica dust (respirable quartz) are metal, coal, and nonmetal (except fuels) mining; foundry, stone clay, and glass production work; and agricultural, chemical production, highway repair, and tuck-pointing work [Akbar-Khanzadeh and Brillhart 2002; Occupational Safety and Health Administration (OSHA) 2004; Rappaport et al. 2003].

Silica dust is an inhalation hazard. Workers may be at risk of silicosis from exposure to silica dust when high-velocity impact shatters the sand into smaller, respirable (< 0.5 to 5.0 μm in diameter) dust particles. Silicosis is a disease where scar tissue forms in the lungs and reduces the ability to extract oxygen from the air. Symptoms of silicosis can be acute, accelerated, or chronic. Acute silicosis may develop within weeks and up to 5 years after breathing large amounts of crystalline silica. Accelerated silicosis may develop shortly after exposure to high concentrations of respirable crystalline silica, whereas chronic silicosis occurs after ≥10 years of exposure to relatively low concentrations of crystalline silica [American Thoracic Society 1997; National Institute for Occupational Safety and Health (NIOSH) 2002]. Many workers in a wider range of industries are exposed to silica, usually in the form of respirable quartz (OSHA 2001, 2003).

OSHA has estimated that more than 2 million workers are exposed to crystalline silica dust in the general, maritime, and construction industries (OSHA 2003). More than 100,000 workers have high-risk exposure to airborne silica dust through construction and mining operations (Akbar-Khanzadeh and Brillhart 2002; NIOSH 1991). There were an estimated 3,600–7,300 newly recognized silicosis cases per year in the United States from 1987 to 1996 (Rosenman et al. 2003). Between 1990 and 1996, 200–300 deaths per year are known to have occurred where silicosis was identified as a contributing cause on death certificates (Akbar-Khanzadeh and Brillhart 2002; OSHA 2003).

The International Agency for Research on Cancer (IARC 1987, 1997) classified crystalline silica as a known human carcinogen. Exposure to crystalline silica has been associated with an increased risk of developing lung cancer (Engholm and Englund 1995; Knutsson et al. 2000; Hughes et al. 2001; Lynge et al. 1986; Robinson et al. 1995; Stern et al. 1995). Previous studies also documented an association between airborne silica exposure and other health problems, including chronic obstructive pulmonary disease, rheumatoid arthritis, scleroderma, Sjogern’s syndrome, lupus, and renal disease (Goldsmith 1997; Hnizdo and Vallyathan 2003; Kane 1997; Parks et al. 2002).

The current OSHA permissible exposure limit (PEL) for crystalline silica is based on a particle counting formula recommended by the ACGIH in the 1970s (ACGIH 1980; OSHA 1989, 1993). In 1986, the ACGIH revised the threshold limit value (TLV) of 0.1 mg/m^3^ for respirable quartz (ACGIH 1986). Currently, the NIOSH (1998) and the ACGIH (2001) both recommend an occupational exposure limit of 0.05 mg/m^3^ for respirable crystalline silica. OSHA recognized the need to revise the PEL to reflect current sampling and analytical methods, and the agency determined to address the significant risk of silicosis and other serious diseases associated with silica through a special emphasis program (SEP) on silicosis (Dear 1996; Jeffress 1998; OSHA 2003).

The purposes of this study were *a*) to summarize measurements of airborne (respirable) crystalline silica dust exposure levels among U.S. workers, *b*) to provide an update of the Stewart and Rice (1990) report on the airborne silica exposure levels in high-risk industries and occupations with data for the time period 1988–2003, *c*) to estimate the number of workers potentially exposed to silica in industries that OSHA inspected for high exposure levels, and *d*) to conduct time trend analyses on silica dust exposure levels for time-weighted average (TWA) measurements.

## Materials and Methods

### Data sources.

The OSHA Integrated Management Information System (IMIS) database was used for the analysis of the airborne concentration of crystalline silica exposure (OSHA IMIS 2003). The OSHA IMIS database contained personal sample measurements of silica exposure (*n* = 11,036) collected during 3,732 OSHA inspections conducted between 1988 and 2003. Of the 11,036 samples, 203 duplicate measures of personal samples were excluded because the number of personal silica samples exceeded the total number of workers who were sampled. A total of 3,188 samples with missing values and 436 area and bulk samples were excluded from the analysis. The remaining 7,209 personal samples collected during 2,512 OSHA inspections were used in this analysis.

### Analytic methods.

The analytic framework used in this study is based on Stewart and Rice’s (1990) method for grouping industries with the highest geometric means and those with the lowest geometric means, where five or more samples were available. We selected a sample size of five arbitrarily as the minimum number required for obtaining stable and reliable descriptive statistics. Personal samples of silica exposure measurements were stratified into two groups by type of inspections to explore if estimates of silica samples were biased in any direction: *a*) all 2,512 inspections and *b*) 948 programmed inspections. Two separate estimation analyses were conducted. First, we analyzed all personal samples (*n* = 7,209) of silica exposure measurements collected during OSHA inspections to determine whether estimates of silica samples collected during complaint, referral, monitoring, follow-up, and fatality inspections were highly biased toward the upper end. Second, we analyzed only personal samples (*n* = 2,868) randomly collected during programmed inspections. In this later analysis, samples collected during complaint, referral, monitoring, follow-up, and fatality inspections were excluded.

In this article, the term “exposure” is defined as the concentration of airborne occupational crystalline silica dust measured in the workers’ personal breathing work environment. In this study we focused on the analysis of personal samples of silica exposure levels measured as an 8-hr TWA measurement among workers in various industries and occupations, and silica levels are expressed as milligrams per cubic meter.

The term “industry” is defined as a group of establishments that primarily engaged in the same kind of economic activity, regardless of their types of ownership. Industries were coded using four-digit Standard Industrial Classification (SIC; Office of Management and Budget 1987) codes. The term “occupation” is defined as a collection of jobs or types of work requiring similar skills, responsibilities, educational requirements, training, licensure and credentials, and the like, found within various industries. To update silica exposure levels among workers with different job titles, the high-risk industries of “stonework masonry” (SIC 1741) and “gray iron foundry” (SIC 3321) with exposure levels above ACGIH TLV of 0.05 mg/m^3^ were selected (Dear 1996). Using 1997 county business patterns (U.S. Census Bureau 1997) and reports to OSHA inspectors by the facility (OSHA IMIS 2003), the percentage and number of workers potentially exposed to crystalline silica by selected industries were estimated.

### Airborne silica measurement.

Personal samples of airborne respirable silica particles were collected using OSHA method ID-142 for quartz in workplace atmosphere using a personal sampling pump and a cyclone assembly (OSHA 1996). Using this method, a respirable sample was collected by drawing air at approximately 1.7 L/min through a 10-mm nylon Dorr-Oliver cyclone attached to a 37-mm diameter polyvinyl chloride filter cassette with a 5-μm pore size (part no. 625413, Mine Safety Appliances, Pittsburgh, PA; or cat. no. P-503700, Omega Specialty Instrument Co., Chelmsford, MA). The cyclone assembly and sampling pump were placed on an employee to collect samples of tiny respirable silica particles from the air in the breathing zone of the employee during an 8-hr work shift. Samples were properly packaged and shipped to the OSHA Salt Lake Technical Center (OSHA 1996). The sample particulates were dissolved in tetrahydrofuran and analyzed by using X-ray diffraction. The qualitative limit of detection for quartz is 5 μg. Further laboratory details are available in OSHA (1996).

### Statistical analyses.

Statistical analyses were conducted using SAS software (SAS Institute, Inc. 1999). First, we conducted a univariate analysis to examine the distribution of the airborne silica exposure levels. We used natural-log transformation of airborne silica exposure levels because silica levels had a positively skewed distribution. In addition to arithmetic mean and median, geometric mean of airborne silica levels and geometric standard deviation (GSD) were calculated for each industry over the period of 1988–2003. Second, the prevalence of elevated crystalline silica exposure levels for 8-hr TWA measurements among workers in high-risk industries and occupations was estimated. Third, a non-parametric regression was applied to make multiple comparisons of silica exposure levels among different major industries, and the null hypothesis of equal variances among different categories of industries and for significant differences in mean exposure levels among industries was tested using *F*-test statistics. Fourth, mixed autoregressive and moving average model [ARMA (1,1)] regression analyses were conducted to evaluate time trends in the silica exposure levels. Finally, a second-order autoregressive error model was created to regress exposure levels on time with errors from one period to be related to errors from the previous two periods. A finding of *p* ≤ 0.05 was considered statistically significant.

The covariates examined for association with higher airborne silica levels were industry, inspections, and year. Industries were grouped into four categories based on the four-digit SIC codes: construction (1521–1799), manufacturing (2011–3299, 3411–3999), metal (3312–3399), and service combined with all other industries including wholesale and retail trade and finance, insurance and real estate, and transportation, communication, and utility (4011–9721) (Office of Management and Budget 1987). Because mining and agricultural industries were not addressed in the OSHA IMIS data, both industries were excluded from this analysis. Dummy variables were used to adjust the significant effect of various industry groups.

## Results

### Prevalence of elevated airborne crystalline silica in occupations and industries.

In the construction industry, “stonework masonry” (SIC 1741) that primarily engages in masonry work, stone cutting, bricklaying, and the like, has been one of the high-risk industries where overexposure to silica exists. Within occupations, the prevalence of elevated airborne silica exposure levels ≥ 0.10 mg/m^3^ among workers with the job title “masonry worker” in the stonework masonry industry was twice as high (6.9%) as the prevalence among workers with the job title “bricklayer” in the same industry (3.1%).

**Figure 1:**
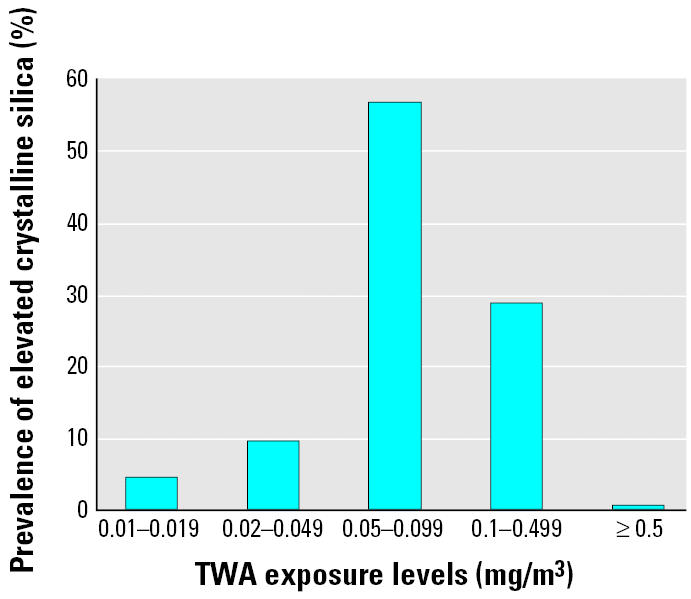
Prevalence of elevated crystalline silica exposure by TWA exposure levels.

The prevalence of elevated airborne crystalline silica exposure levels ≥ 0.50 mg/m^3^ was 0.5% (*n* = 36) for all sampled workers (Figure 1). The proportion of workers with elevated airborne silica exposure levels ≥ 0.10 mg/m^3^ was 29.9% (*n* = 2,106). Within industries, workers in the metal industry had a prevalence of elevated airborne silica exposure levels ≥ 0.05 mg/m^3^ (35.6%), 2.9 times higher than the prevalence among workers in the construction industry (12.4%).

### Airborne crystalline silica dust levels.

Table 1 presents arithmetic mean, geometric mean, standard deviations, and median of 8-hr TWA exposure measurements by industries with the highest and lowest airborne silica exposure. Geometric mean (GSD) airborne silica exposure levels were between 0.017 mg/m^3^ (GSD, 0.931 mg/m^3^; surgical appliances supplies industry, SIC 3842) and 0.166 mg/m^3^ (GSD, 0.943 mg/m^3^; metal valves and pipe fitting industry, SIC 3494). The geometric mean and GSD airborne silica exposure levels by industries and type of inspections are shown in Table 2. The overall geometric mean of silica exposure levels for samples collected during programmed inspections was 0.077 mg/m^3^. The geometric mean of samples collected under all inspections combined was higher in eight industries, whereas the geometric mean from programmed inspections was higher in two industries (Table 2).

Table 3 presents the airborne silica exposure levels by occupations in the “gray iron foundry” industry (SIC 3321). Gray iron foundry is the industry that primarily engages in manufacturing gray and ductile iron castings, including cast iron pressure and soil pipes and fittings. Workers with the job title “spruer” had the highest geometric mean airborne silica exposure levels (0.154 mg/m^3^), followed by workers with the job title “hunter operator” (0.093 mg/m^3^), those with the job title “charger” (0.091 mg/m^3^), and workers with the job title “core maker” (0.078 mg/m^3^).

The airborne silica exposure levels by occupations in the “stonework masonry” industry (SIC 1741) are shown in Table 4. The overall geometric mean of silica exposure levels for workers in this industry was 0.065 mg/m^3^. The geometric mean silica exposure levels were highest in those workers with the job title “helper” (0.099 mg/m^3^), followed by those with the job title “stone cutter” (0.070 mg/m^3^), those with the job title “bricklayer” and “laborer” (0.067 mg/m^3^), and workers with the job title “masonry worker” (0.065 mg/m^3^).

There were an estimated 119,381 workers potentially exposed to crystalline silica in 18 selected industries (Table 5). An estimated 25,027 workers were potentially exposed to airborne silica exposure in the automotive repair paint shop (SIC 7532) compared with 114 workers in the metal valves and pipe fitting industry (SIC 3494). Workers potentially exposed to silica exposure in stonework masonry (SIC 1741), plastering drywall work (SIC 1742), and tile, marble, and mosaic work (SIC 1743) were estimated at 44,989 employees. Workers in the testing laboratories services (SIC 8734) were estimated at 18,497 potentially exposed to airborne silica exposure.

The nonparametric regression showed the mean square error (MS) in airborne silica exposure between industries (MS_bi_ = 0.048) and within industries (MS_wi_ = 0.014), with *F* (3, 7,205) = 3.28 (*p* = 0.02). In this analysis we rejected the null hypothesis of no significant differences in the mean exposure levels between industries. We attempted to fit a mixed autoregressive and moving average model, ARMA (1,1), to the silica exposure data. A chi-squared value of 12.6 (*p* = 0.01) showed that we could not reject the hypothesis that the residuals are correlated. Thus, ARMA (1,1) was not an adequate model for silica exposure data. A final second-order autoregressive error model showed that a decline in the airborne silica exposure levels of 10.0% was observed per year between 1988 and 2003, but it was not statistically significant (*p* = 0.18, *R*^2^ = 0.0398). Within industries, the autroregressive error model AR(2) predicted that the construction industry has significantly lower airborne silica exposure levels (*p* = 0.10) during this time period. The findings also predicted that manufacturing industries have higher silica exposure levels than the metal industries, but it was not statistically significant at *p* ≤ 0.05. The estimated autocorrelation coefficients ρ_1_ and ρ_2_ were −0.153 and −0.082, respectively, with an estimated variance of error term of 0.014. The results showed that the negative effect of an OSHA inspection on the airborne silica exposure levels was estimated at β = −0.007, with *p* = 0.0319.

## Discussion

Our findings suggest that geometric mean crystalline silica exposure levels have declined in some high-risk construction industries during 1988–2003. A comparison of our results with silica exposure levels found in a previous study by Stewart and Rice (1990) revealed a significant decline over the years. The geometric mean airborne silica exposure level in the general contractor industry (SIC 1541) was almost 6.2 times higher, at 0.354 mg/m^3^ (Stewart and Rice 1990), in 1979–1987 compared with 0.057 mg/m^3^ in 1988–2003. The geometric mean airborne silica exposure levels in the bridge tunnel construction industry (SIC 1622) were 5.5 times higher, at 0.383 mg/m^3^, in 1979–1987 compared with 0.069 mg/m^3^ in 1988–2003. The stonework masonry industry (SIC 1741) had geometric mean airborne silica exposure levels 9.8 times higher, at 0.619 mg/m^3^, in 1979–1987 than its level, 0.063 mg/m^3^, in 1988–2003. The significant decline of airborne silica observed in the construction industry could be explained by the implementation of advanced health and safety programs, effective engineering controls, work practice controls, and personal protective equipment (Flanagan et al. 2003; Flynn and Susi 2003).

Silica exposure levels among workers in the gray iron industry (SIC 3321) were significantly lower in 1988–2003 than in 1979–1987. Our results also showed that silica exposure levels for workers with the job title “furnace operators” declined by 53.5% of what they were in 1979–1987, from 0.142 mg/m^3^ (Stewart and Rice 1990) to 0.066 mg/m^3^. Geometric mean airborne silica exposure levels for workers with the job title “grinder” went down by 28.6%, from 0.105 mg/m^3^ to 0.075 mg/m^3^. Furthermore, silica exposure levels for workers with the job title “reline cupola” decreased more than 5.7 times, from 0.384 mg/m^3^ in 1979–1987 (Stewart and Rice 1990) to 0.067 mg/m^3^ in 1988–2003. Geometric mean silica exposure levels for workers with the job title “cleaning department” declined by 50.8%, from 0.122 mg/m^3^ to 0.060 mg/m^3^, whereas exposure levels for workers with the job title “sorter” decreased from 0.127 mg/m^3^ (Stewart and Rice 1990) to 0.067 mg/m^3^.

The recent decline of airborne silica exposure levels in the gray iron foundry could be attributed to many potential factors, in addition to OSHA’s enforcement as part of its SEP for workplace exposure to silica (Jeffress 1998). Because of the OSHA inspections and enforcement actions, most foundry industries were required to take action to reduce the overexposure and comply with OSHA’s standard PEL (Irwin 2003). The OSHA PEL was defined by a formula that included the percentage respirable silica (OSHA 2001). Assuming that the dust is 100% crystalline silica, the OSHA PEL is computed at 0.1 mg/m^3^. Using the OSHA-calculated PEL of 0.436 mg/m^3^ as the criterion, 3.6% of the sampled workers were overexposed to airborne silica exposure, whereas using the ACGIH TLV of 0.05 mg/m^3^ as the criterion, 85.5% of the sampled workers were overexposed (Figure 1). An overexposure severity factor was defined when the TWA exposure level was divided by the OSHA-calculated PEL. The overexposure severity factor was estimated at 0.17, less than 1. Our findings were eight times lower than Galster’s (1997) findings of 30% of air samples over the OSHA PEL.

Our estimates of the number of workers exposed were consistent with an earlier study done by Linch et al. (1998), which reported approximately 132,000 workers in the construction industry with three-digit SIC code 174 to be potentially exposed to airborne silica during the 1981–1983 national hazard survey. The results of this study suggest that the number of workers potentially exposed to crystalline silica in the construction industry with SIC codes 1741, 1742, and 1743 combined was almost three times (44,989 workers) lower in 2003 than it was in 1981–1983 (Table 5). Linch et al. (1998) also reported that an estimated 41,700 workers in the research testing services industry with a three-digit SIC code 873 were exposed to airborne silica at least twice the NIOSH-recommended exposure limit using the 1993 IMIS database. The number of workers exposed in the testing laboratories services industry (SIC 8734) has declined more than 2-fold in the last decade, from 41,700 in 1993 to 18,497 in 2003.

**Figure 2:**
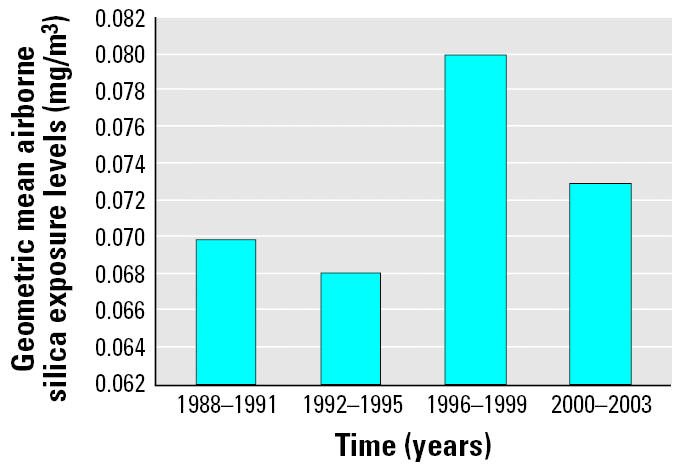
Geometric mean airborne silica exposure levels by year.

Although the airborne silica exposure levels declined in some industries and processes, the results showed an upward trend in the silica respirable dust exposure levels in certain industries and occupations, and exposure levels were above the ACGIH TLV of 0.050 mg/m^3^ (ACGIH 2001). For instance, in the gray iron foundry industry (SIC 3321), exposure levels for workers with the job title “spruer” increased from 0.098 mg/m^3^ (Stewart and Rice 1990) in 1979–1987 to 0.154 mg/m^3^ in 1988–2003, an increase of 57.1%. Airborne silica exposure levels went up from 0.068 mg/m^3^ in 1992–1995 to 0.080 mg/m^3^ in 1996–1999 (Figure 2). Because many businesses are not yet in compliance with OSHA health standards, large numbers of workers in certain industries and occupations continue to be exposed to silica dust in the course of their work (Flanagan et al. 2003).

The model of the second-order autoregressive error showed significant association between airborne silica exposure levels and OSHA inspections. Using *R*^2^ as a measure of “goodness of fit,” 3.98% of the total variation in airborne silica exposure levels was explained by the model. This low *R* ^2^ might be due to the lack of data on other explanatory variables that should be included in the model. Future research is needed to further examine other potential predictors in explaining the variability of airborne silica exposure levels.

Almost two-fifths of all inspections conducted by OSHA are programmed inspections. In programmed inspections, OSHA may identify industries with the greatest risk of injury and illness to workers, and then target firms sampled randomly within them. For general and construction industry, inspections initiated under the SEP are required to be programmed (scheduled) and conducted in accordance with the provisions in the Field Inspection Reference Manual (FIRM) and the Revised Field Operations Manual (FOM) (OSHA 1994, 1995; Dear 1996). Wherever possible, inspections focus on particular establishments where overexposures to airborne silica are most likely or there are known cases of silicosis (Dear 1996). When making an inspection, OSHA takes sampling exposure measurements of employees who may have high or low exposure over an 8-hr TWA. However, all inspections involving fatalities, complaints, follow-up, or referrals tend to have a potential bias toward high estimates of exposure levels (Linch et al. 1998). In this study, it was observed that the geometric mean of samples collected during all inspections combined was higher in eight industries than the mean of samples collected during programmed inspections (Table 2).

This study has some limitations. First, OSHA samples measure the workers’ personal breathing work environment exposure without taking into account the use of a respirator. Actual exposure levels for some workers may be much less than the workers’ ambient readings of exposure. As a result, this sampling measurement may overestimate the workers’ exposure levels. Inferences regarding OSHA inspections must be interpreted with caution, especially in cases of small sample sizes. Second, a potential limitation of the IMIS database is its inability to identify the duration of employment of the individual worker and the duration of exposure to silica dust. Third, SIC codes were used in the classification of establishments by type of primary activity in which they were engaged. For industries with multiple activities, it is possible that one may classify an industry by its processes rather than products manufactured. Fourth, job titles in the IMIS database were not well defined and coded according to a common and standardized system. Because of this lack of common classification codes, it may be necessary to categorize job titles and aggregate them into fewer categories. Finally, because the IMIS database does not represent a random sample of exposure levels, the findings of this study may not be generalizable. Nonetheless, these limitations are not serious enough to invalidate the findings of this study.

The strength of the OSHA IMIS database is its ability to provide estimates of airborne silica exposure levels and to identify high-risk industries and occupations. It is the largest source of occupational exposure data. As long as the limitations of the OSHA IMIS data set are understood, it provides an important source of information regarding occupational exposure. It also may provide a useful tool to generate hypotheses that could be tested in future studies.

## Conclusions

Although occupational exposure to crystalline silica dust levels declined in some industries and occupations, the results showed that workers in certain industries and occupations were still overexposed. Approximately 3.6% of the sampled workers were overexposed to airborne silica above the OSHA-calculated PEL. OSHA regulatory efforts are needed to further increase industry compliance with occupational exposure limits by enforcing effective engineering controls and to protect workers from overexposure to crystalline silica. Furthermore, OSHA needs to increase its compliance assistance and outreach efforts to assist businesses in establishing programs to reduce overexposure to silica.

## Figures and Tables

**Table 1:** Arithmetic mean (AM), geometric mean (GM), their standard deviations (ASD, GSD), and median of exposure measurements of crystalline silica (mg/m^3^) by industry, IMIS (1988–2003).

Industry*^a^* (SIC code)	No.*^b^*	AM	ASD	GM	GSD	Median
Metal valves and pipe fitting (3494)	8	0.229	0.161	0.166	0.943	0.243
Industrial supplies (5085)	5	0.175	0.090	0.161	0.431	0.147
Roofing siding and sheet metal (1761)	11	0.224	0.170	0.150	1.029	0.230
Special industry machinery (3559)	15	0.193	0.167	0.127	0.978	0.110
Automotive repair paint shop (7532)	13	0.161	0.143	0.107	0.968	0.050
Mining machinery equipment (3532)	10	0.080	0.075	0.046	1.323	0.050
Plastics plumbing fixtures (3088)	14	0.054	0.033	0.044	0.682	0.050
Plastering drywall work (1742)	13	0.045	0.046	0.031	0.920	0.022
Tile, marble, and mosaic work (1743)	12	0.036	0.027	0.025	0.958	0.035
Surgical appliances supplies (3842)	5	0.024	0.019	0.017	0.931	0.018

a The 10 industries with the highest and lowest geometric mean where at least five samples were available.

b Number of personal TWA sample measurements.

**Table 2:** Geometric mean (GM) and GSD of exposure measurements of crystalline silica (mg/m^3^) by industry and type of inspection, IMIS (1988–2003).

	All inspections (*n* = 2,512)				Programmed inspections (*n* = 948)						
Industry*^a^* (SIC code)	No.*^b^*	GM	GSD	No.*^b^*	GM	GSD
Soap and other detergents (2841)	6	0.102	0.757	5	0.108	0.831
Testing laboratories services (8734)	53	0.099	0.896	19	0.082	0.656
Cut stone and stone products (3281)	405	0.091	0.956	164	0.075	0.963
General contractors (1541)	28	0.091	0.900	8	0.057	0.346
Coating engraving (3479)	75	0.075	0.839	26	0.072	0.842
Gray iron foundries (3321)	1,760	0.073	0.877	782	0.082	0.899
Concrete work (1771)	94	0.073	0.705	38	0.072	0.720
Manufacturing explosives (2891)	9	0.070	0.841	5	0.058	0.581
Bridge tunnel construction (1622)	91	0.070	0.827	41	0.069	0.761
Stonework masonry (1741)	274	0.065	0.732	111	0.063	0.803
All	7,209	0.073	0.919	2,868	0.077	0.935

a The industries where at least five samples were collected during inspections.

b Number of personal TWA sample measurements.

**Table 3:** Arithmetic mean (AM), geometric mean (GM), their standard deviations (ASD, GSD), and median of exposure measurements of crystalline silica (mg/m^3^) by occupation in the gray iron foundry industry (SIC 3321), IMIS (1988–2003).

Occupation	No.*^a^*	AM	ASD	GM	GSD	Median
Spruer	22	0.232	0.182	0.154	0.100	0.205
Hunter operator	10	0.157	0.151	0.093	1.144	0.050
Charger	8	0.146	0.156	0.091	0.999	0.050
Core maker	89	0.129	0.135	0.078	1.033	0.050
Grinder	371	0.112	0.123	0.075	0.821	0.050
Molder	308	0.116	0.129	0.073	0.910	0.050
Abrasive blast operator	56	0.103	0.110	0.070	0.821	0.050
Sorter	23	0.098	0.108	0.067	0.827	0.050
Reline cupola	29	0.096	0.113	0.067	0.725	0.050
Furnace operator	47	0.096	0.110	0.066	0.766	0.050
Core setter	23	0.086	0.082	0.066	0.671	0.051
Craneman	16	0.097	0.106	0.066	0.815	0.050
Cleaning department	36	0.094	0.117	0.060	0.879	0.050
Inspector	21	0.118	0.146	0.057	1.298	0.050
Ladle repair	30	0.081	0.098	0.055	0.829	0.050

a Number of personal TWA sample measurements.

**Table 4:** Arithmetic mean (AM), geometric mean (GM), their standard deviations (ASD, GSD), and median of exposure measurements of crystalline silica (mg/m^3^) by occupation in the stonework masonry industry (SIC code 1741), IMIS (1988–2003).

Occupation	No.*^a^*	AM	ASD	GM	GSD	Median
Helper	6	0.175	0.198	0.099	1.143	0.050
Stone cutter	33	0.097	0.096	0.070	0.814	0.050
Bricklayer	30	0.091	0.086	0.067	0.742	0.050
Laborer	48	0.093	0.102	0.067	0.731	0.050
Masonry worker	74	0.088	0.090	0.065	0.713	0.050
Foreman	8	0.085	0.081	0.064	0.748	0.050
Tuckpointer	18	0.086	0.110	0.062	0.647	0.050
Grinder	35	0.055	0.020	0.052	0.372	0.050
Hod carrier	5	0.092	0.123	0.042	1.540	0.050
All	257	0.088	0.093	0.065	1.140	0.050

a Number of personal TWA sample measurements.

**Table 5:** Estimates of the number and percentage of workers potentially exposed to crystalline silica by selected industries, IMIS (1988–2003).

Industry*^a^* (SIC code)	No.*^b^* of workers in the establishment	Percent of workers exposed*^c^*	Total no. of potentially exposed workers*^d^*
Metal valves and pipe fittings (3494)	18,080	0.63	114
Special industry machinery (3559)	111,312	0.56	623
Automotive repair paint shop (7532)	205,906	12.2	25,027
Soap and other detergents (2841)	30,352	1.4	438
Testing laboratories services (8734)	82,786	22.3	18,497
Gray iron foundries (3321)	82,749	1.7	1,395
Manufacturing explosives (2891)	21,322	5.3	1,131
Fabricated rubber products (3069)	56,079	1.2	698
Masonry, stonework (1741)	168,155	12.7	21,302
Brick, stone, related material (5032)	34,241	6.4	2,203
Repair shops, NEC (7699)	212,049	8.0	17,022
Transmission equipment (3568)	20,884	2.1	438
Chemical preparations, NEC (2899)	34,873	7.9	2,766
Mining machinery equipment (3532)	13,631	2.4	329
Plastics plumbing fixtures (3088)	16,793	15.9	2,670
Plastering drywall work (1742)	262,530	4.8	12,459
Tile, marble, and mosaic work (1743)	38,051	29.5	11,228
Surgical appliances supplies (3842)	96,154	1.1	1,041
Total	1,505,947	7.9	119,381

NEC, not elsewhere classified.

a Industries with the highest and lowest geometric mean where at least five samples were available.

b Number of workers in the establishments, as reported to the U.S. Census Bureau (1997)

c Percentage of workers exposed was calculated by dividing the number of workers exposed as determined by the inspector, and the number of workers in the establishment, as reported to the OSHA inspector by the facility.

d Total number of potentially exposed workers in an SIC was calculated by taking the product of the proportion of workers exposed in each SIC by the average worker population employed nationally in each SIC, as reported to the U.S. Census Bureau (1997).
